# Effect of the rho-kinase inhibitor ripasudil in needling with mitomycin C for the failure of filtering bleb after trabeculectomy: a cross-sectional study

**DOI:** 10.1186/s12886-022-02680-9

**Published:** 2022-11-14

**Authors:** Yu Mizuno, Naoki Okada, Hiromitsu Onoe, Kana Tokumo, Hideaki Okumichi, Kazuyuki Hirooka, Yoshiaki Kiuchi

**Affiliations:** Department of Ophthalmology and Visual Science, Hiroshima University, 1-2-3 Kasumi Minamiku, Hiroshima, 734-8551 Japan

**Keywords:** Needling, Ripasudil, Rho-associated protein kinase inhibitor, Trabeculectomy, Glaucoma, Mitomycin C

## Abstract

**Background:**

Rho-kinase inhibitors can inhibit fibrosis after glaucoma surgery. This study aimed to evaluate the effect of rho-kinase inhibitor after needling procedure with mitomycin C for the failure of filtering bleb with trabeculectomy.

**Methods:**

This retrospective single-center study examined the effects of rho-kinase inhibitor after the needling procedure. We included 27 eyes of 27 patients with glaucoma who underwent needling procedure using mitomycin C and were subsequently treated with ripasudil—a rho-associated protein kinase inhibitor (ripasudil group)—or without ripasudil (control group). The ripasudil and control groups were compared in terms of intraocular pressure (IOP) and the number of antiglaucoma medications. Success at 12 months after the needling procedure was defined as a > 20% decrease in IOP from the preoperative period without surgical reintervention.

**Results:**

At 12 months after the needling procedure, the mean IOP decreased from 16.9 ± 4.5 to 12.6 ± 1.1 mmHg in the control group and from 16.0 ± 5.3 to 12.2 ± 1.2 mmHg in the ripasudil group (*p* = 0.77). The 12-month success rates were 60.00% and 56.25% in the control and ripasudil groups (*p* = 0.98), respectively. In the preoperative period, the numbers of antiglaucoma drugs were 0.27 ± 0.46 and 0.92 ± 0.91 in the control and ripasudil groups (*p* = 0.022), respectively, and at 12 months after the needling procedure, they were 1.07 ± 1.44 and 0.73 ± 1.10 (*p* = 0.52), respectively.

**Conclusions:**

Treatment with ripasudil (a rho-associated protein kinase inhibitor) after the needling procedure with mitomycin C did not show better results than treatment with the mitomycin C needling procedure alone at 12 months after the procedure.

## Background

Glaucoma is an optic neuropathy characterized by gradual progressive morphological changes in the optic disc and visual field loss [1]. In patients with glaucoma, trabeculectomy is an effective surgical technique for slowing the progression of visual field loss by lowering intraocular pressure (IOP) [2]. It creates a drainage pathway between the anterior chamber and the sub-Tenon space, creating a subconjunctival space for the aqueous humor, referred as filtrating bleb. The success of trabeculectomy depends on the continuous passage of aqueous humor between the anterior chamber and the subconjunctival space. However, fibroblast proliferation and scar formation under the conjunctival and episcleral interface of the filtrating bleb are the most common causes of trabeculectomy failure [3, 4]. The transconjunctival bleb needling procedure is designed to rebuild failing blebs by mechanically stripping the adhesions. To prevent fibroblast proliferation, antimetabolites, such as 5-fluorouracil (5-FU) or mitomycin C (MMC), have been injected subconjunctivally in the bleb needling procedure [5–7].

A newer antiglaucoma medication, ripasudil (Glanatec®, ophthalmic solution 0.4%, Kowa Company, Ltd., Japan), which is a rho-associated protein kinase (ROCK) inhibitor ophthalmic solution, was approved in Japan in 2014 for the treatment of glaucoma and ocular hypertension. The mechanism for lowering IOP involves changing the status of the trabecular meshwork and Schlemm’s canal endothelial cells, resulting in an elevation of the conventional aqueous outflow [8–12]. The safety of ripasudil is well established. Several in vitro rabbit model study reports have shown that topical treatment with the ROCK inhibitor Y-27632 was able to prevent excessive scarring after glaucoma filtration surgery [13, 14]. However, there is no consensus on the clinical use of the ROCK inhibitor ripasudil to prevent scarring after needling procedures in humans. In this study, we evaluated the results of patients with glaucoma who underwent needling procedure with MMC compared with those receiving MMC and ripasudil ophthalmic solution for the prevention of scarring after the procedure.

## Methods

In this retrospective comparative study, we evaluated patients who attended the Hiroshima eye clinic in Japan between September 2019 and January 2021. The study received approval from the Ethical Committee for Epidemiology of Hiroshima University (protocol code E—2142, August 6, 2020), and the research adhered to the tenets of the Declaration of Helsinki.

We analyzed 27 eyes of 27 patients with glaucoma who underwent the bleb needling procedure with MMC due to trabeculectomy failure with a fornix-based conjunctival flap using MMC. None of the patients had any intraoperative complications during the needling procedure.

We included only patients who underwent the bleb needling procedure for the first time after trabeculectomy with MMC. For the ripasudil group, we included 12 eyes of 12 patients who received a one-drop instillation of ripasudil into the eye twice a day for at least 3 months after the bleb needling procedure with MMC. Fifteen eyes of 15 patients who had undergone the bleb needling procedure with MMC but did not receive ripasudil instillation treatment were recruited during a similar period (control group). The exclusion criteria were patients who underwent any ocular surgery after the last trabeculectomy. All patients underwent an ophthalmic examination, and the following data were collected: age, sex, type of glaucoma, preoperative and postoperative IOPs (Goldmann Applanation Tonometer, Haag-Streit, Köniz, Switzerland), lens status, period between the last trabeculectomy and needling procedure, and number of antiglaucoma medications. Preoperative and postoperative IOPs were defined as IOPs measured on the day before and after the needling procedure, respectively.

### Needling procedure

All needling procedures were performed in an outpatient department. After the administration of topical anesthesia (0.4% oxybuprocaine and 0.1% adrenaline), we applied iodine and polyvinyl alcohol (PA·IODO Ophthalmic and Eye washing solution diluted six times with saline solution) to the external eye. Subsequently, we injected 0.1 mL of 2% xylocaine with epinephrine and 0.1 mL of 0.04% MMC using a 27-gage needle at the subconjunctiva approximately 10 mm distal to the bleb, as described in a previous study [15]. After 5 min, we inserted a 27-gage needle or bleb knife (Kai Medical, Tokyo, Japan) at the same site. The needle or bleb knife was introduced under the scleral flap, and the fibrotic tissues were then cut and raised. After the procedure, patients were instructed to use topical antibiotics (1.5% levofloxacin) and anti-inflammatory (0.1% betamethasone sodium phosphate or 0.1% fluorometholone ophthalmic suspension) eye drops four times daily from 1 month to 1 year. In the ripasudil group, the use of ripasudil twice daily for least 3 months was added.

To evaluate the efficacy of the treatments, we defined success as a > 20% reduction in IOP from preoperative IOP. An IOP that was higher than the specified criterion for two consecutive measurements was considered a failure from the first time point at which the IOP did not meet the criterion. Cases that required a re-needling procedure or another glaucoma surgery were classified as failures. We analyzed the success rate according to the defined criteria.

### Statistical analysis

Data were entered into an Excel spreadsheet (Microsoft Corp., Redmond, WA, USA). We performed all statistical analysis using JMP pro software (version 16, SAS, Inc., Cary, NC, USA).

The sample size was calculated based on a previous study [7]. The response within each subject group was normally distributed with standard deviation 1.5. If the true difference in the experimental and control means was 1.8, we would need to include 12 patients in each group to be able to reject the null hypothesis that the population means of the ripasudil and control groups are equal with a probability power of 0.8. The Type I error probability associated with the test of this null hypothesis is 0.05. Measurement data were expressed as the mean ± standard deviation with a 95% confidence interval. Continuous data from the ripasudil and control groups were analyzed using Student’s t-test, whereas discrete data were analyzed using Pearson’s chi-square test. Differences were statistically significant when the *p* value was < 0.05. We compared the success rates for each group using Kaplan–Meier survival analysis and log-rank test. We analyzed the risk factors for needling procedure failure with MMC using Cox proportional hazards models.

## Results

We analyzed 27 eyes of 27 patients with glaucoma who underwent bleb needling procedure with MMC due to trabeculectomy failure with a fornix-based conjunctival flap using MMC. Table 1 presents a summary of the demographic data.

**Table 1:** Demographic and preoperative characteristics

	Control (*n* = 15)	Ripasudil (*n* = 12)	*p*
Mean preoperative IOP (mmHg)	16.9 ± 4.5	16.0 ± 5.3	*0.62*
Mean number of antiglaucoma medications	0.27 ± 0.46	0.92 ± 0.91	*0.022*
Age (years)	67.67 ± 12.22	63.92 ± 19.57	*0.55*
Gender (male/female)	7/8	4/8	*0.48*
Laterality (right/left)	7/8	6/6	*0.86*
Glaucoma classification			*0.31*
POAG	11	7	
PACG	0	1	
Exfoliation G	3	1	
Secondary G	1	3	
Time from TLE surgery to needling procedure (months)	24.36 ± 49.49	34.39 ± 28.87	*0.54*
Lens status			*0.66*
Phakia	5	5	
IOL	10	7	

*IOP* Intraocular pressure, *POAG* Primary open-angle glaucoma, *PACG* Primary angle-closure glaucoma, *G* Glaucoma, *TLE* Trabeculectomy, *MMC* Mitomycin C, *IOL* Intraocular lens

**Fig. 1:**
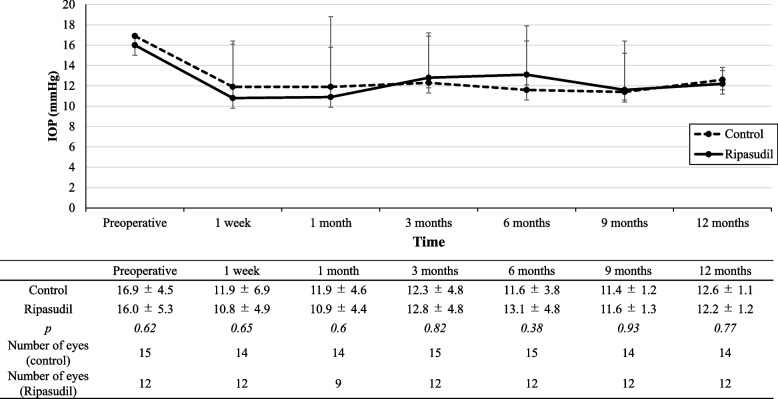
Preoperative and postoperative mean IOPs (mmHg). Intraocular pressure (IOP) of the control (solid line) and ripasudil (dotted line) groups before needling and at each time after the needing procedure. Error bars show standard deviation (SD). There was no difference between the two groups at each time (Student’s t-test)

The preoperative characteristics were similar in the both groups. However, before the needling procedure, the mean number of antiglaucoma medications was higher in the ripasudil group than that in the control group (0.92 ± 0.91 and 0.27 ± 0.46, respectively; *p* = 0.022). The period from trabeculectomy to the first needling procedure with MMC was 24.36 ± 49.49 months (range, 0.53–195.63 months) and 34.39 ± 28.87 months (range, 3.63–92.3 months) in the control and ripasudil groups (*p* = 0.54), respectively. Figure 1 presents the mean preoperative and postoperative IOPs (Fig. 1).

The mean preoperative IOP was 16.9 ± 4.5 and 16.0 ± 5.3 mmHg in the control and ripasudil groups, respectively, which decreased to 12.6 ± 1.1 and 12.2 ± 1.2 mmHg (reduction of 25.34% and 23.88%), respectively.

**Fig. 2:**
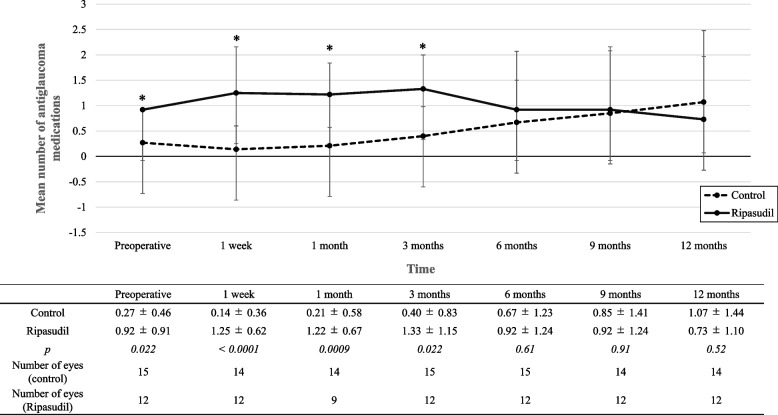
Preoperative and postoperative mean numbers of antiglaucoma medications. The mean number of antiglaucoma medications of the control (solid line) and ripasudil (dotted line) groups before needling and at each time after the needing procedure. Error bars show standard deviation (SD). *Comparison between the two groups (*p* < 0.05, Student’s t-test)

**Fig. 3:**
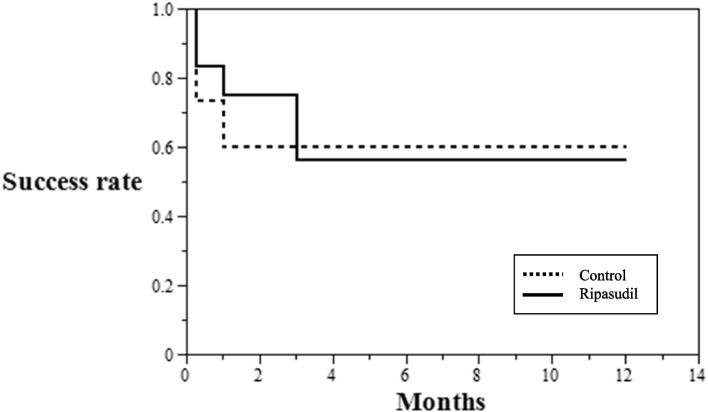
Kaplan–Meier survival curve. The 12-month survival rate was 60.00% and 56.25% in the control and ripasudil groups, respectively (*p* = 0.98, log-rank test)

The preoperative and postoperative mean numbers of antiglaucoma medications are presented in Fig. 2. In the ripasudil group, ripasudil was continued for 3 months after the needling procedure and then continued or terminated at the discretion of the attending surgeon, or it could be changed to another type of antiglaucoma medication. None of the patients stopped ripasudil because of side effects for at least 3 months. This might be why the number of antiglaucoma medications in the ripasudil group was higher than that in the control group during the first 3 months. In the preoperative period, the mean number of antiglaucoma medications was higher in the ripasudil group than that in the control group. However, at 12 months after the needling procedure, there was no significant difference in the mean number of antiglaucoma medications between the ripasudil and control groups (0.73 ± 1.10 and 1.07 ± 1.44, respectively; *p* = 0.52). On the basis of the Kaplan–Meier survival plot, the 12-month survival rates were 60.00% and 56.25% in the control and ripasudil groups, respectively (*p* = 0.98; see Fig. 3).

We analyzed the risk factors for needling procedure failure with MMC at 12 months after the needling procedure using Cox proportional hazards models to estimate the hazard ratio and 95% confidence interval (see Table 2).

**Table 2:** Hazard ratio for needling procedure success after 12 months

Study factor	Category	Hazard Ratio	95% Confidence Interval	*p*
Group	Control	1		
	Ripasudil	1.26	0.25–6.47	*0.78*
Age	< 65 years	1		
	≥ 65 years	0.37	0.07–4.33	*0.24*
Lens status	Phakia	1		
	IOL	1.02	0.24–4.33	*0.98*
Period between trabeculectomy to needling	< 4 months	1		
	≥ 4 months	0.26	0.03–2.11	*0.21*
IOP before needling	< 18 mmHg	1		
	≥ ≧18 mmHg	0.70	0.15–3.22	*0.65*
Number of antihypertensive medications before needling	< 1	1		
	≥ 1	2.16	0.27–17.12	*0.47*
Instilled ripasudil before needling	Ripasudil	1		
	Other or none	3.64	0.38–34.87	*0.26*
Anti-inflammatory eye drops after needling	Betamethasone	1		
	Fluorometholone	3.15	0.31–31.59	*0.33*

*IOL* Intraocular lens, *IOP* Intraocular pressure

We used several variables (use of ripasudil after needling, age, lens status, period between trabeculectomy and needling, IOP before needling, number of antiglaucoma medications before needling, instilled ripasudil before needling, and anti-inflammatory eye drops after needling) for the multivariable Cox proportional hazards regression analysis. We found no statistically significant association in the multivariable analysis. Surgical reinterventions were required in five eyes. In the control group, two eyes needed re-needling (13.3%), whereas in the ripasudil group, two eyes needed re-needling and one eye needed bleb revision (25.0%).

## Discussion

This midterm study showed that the addition of ripasudil after the needling procedure with MMC did not improve surgical outcomes in terms of IOP.

The bleb needling procedure is a useful intervention for failed bleb after trabeculectomy. Although the techniques for needle revision vary, the basic procedure is performed by lysing or puncturing bands of fibrotic tissues to increase filtration [16].

During glaucoma filtration surgery, the scleral flap or conjunctiva is exposed to cytokines and growth factors that are produced by various types of inflammatory cells and fibroblasts [17]. These responses activate the migration of fibroblasts and other inflammatory cells. Activated fibroblasts are produced and secreted via the extracellular matrix as collagen for fibrosis. ROCK inhibitor has been reported to suppress the expression of extracellular matrix degrading enzymes in fibroblasts and to decrease extracellular matrix secretion [18]. Diah et al. indicated that the ROCK inhibitor Y‐27,632 may inhibit fibrosis and improve outcomes after glaucoma filtration surgery by inhibiting the transdifferentiation of Tenon fibroblasts into myofibroblasts and the transforming growth factor-β and mitogen-activated protein kinase signaling after surgery [19]. Honjyo et al. also reported that, in a rabbit model, the ROCK inhibitor Y-27632 inhibited wound healing and fibroproliferation after filtration surgery [13].

On the basis of these properties, our study presents a clinical retrospective examination of the addition of the ROCK inhibitor ripasudil for clinically reducing subconjunctival scarring after the first needling procedure with MMC. We found no significant difference in IOP reduction and success rates between the control and ripasudil groups. The number of antiglaucoma medications, which was greater preoperatively in the ripasudil group than in the control group (0.92 ± 0.91 and 0.27 ± 0.46, respectively; *p* = 0.022), became equivalent at 12 months after needling procedure. These results might indicate that ripasudil suppressed fibroproliferation after the needling procedure.

We also investigated the predictors of needling procedure success; however, no specific factors were identified in our study. Several factors have been identified as associated with needling procedure failure, including young patient age [20], fornix-based conjunctival flap trabeculectomy [21], lack of MMC use during the previous filtration surgery [22], and higher IOP before the needling procedure [22, 23]. In our study, all eyes received fornix-based conjunctival flap trabeculectomy using MMC. Regarding the risk factors of needling procedure failure, Gutiérrez-Ortiz et al. reported that a needling procedure performed less than 4 months after trabeculectomy was highly correlated with the success rate [24]. Additionally, Swan reported that a short interval between the initial surgery and needling procedure resulted in a reduced rate of needling procedure success [25]. Although we noted a variation in the time elapsed between trabeculectomy and the first needling procedure in our study, the period between trabeculectomy and needling procedure with MMC was not a factor affecting the success in either of the groups.

Our study has several limitations. First, our study used a retrospective, nonrandomized design and had a limited sample size. The small size of the groups greatly reduced the power of this study to detect differences. Second, as Mimura et al. reported that ripasudil can increase the success rate of TLE without MMC in patients with uveitic glaucoma [26], the use of MMC in the needling procedure may mask the result. The use of antifibrotic agents, such as MMC or 5-FU, may be important not only in trabeculectomy but also in needling procedures [27, 28]. Wound healing mechanisms can easily cause the conjunctival fibrosis to close the new opening hole made by the needle. There is evidence that the success rates in trabeculectomy are higher with antifibrotic agents than without, although further research is needed to determine whether MMC or 5-FU is more effective in maintaining the bleb to lower IOP. The use of bleb needling procedure using subconjunctival antimetabolites, such as MMC or 5-FU, has recently become widespread. There are several reports that bleb needling procedure with antimetabolites may increase IOP control or reduce the use of antiglaucoma medications [29, 30]. In our study, we used MMC in the needling procedure in all cases, which has a strong effect on antimetabolic activity and suppresses the wound healing response; therefore, potentially covering up the effect of ROCK inhibitor after the needling procedure. A further prospective randomized controlled trial with appropriate sample is needed to clarify the effect of ROCK inhibitor after the needling procedure with and without MMC. Third, the difference in the number and contents of antiglaucoma medications before and 3 months after the needling procedure and the difference in the contents of anti-inflammatory eye drops after the needling procedure between the groups may indicate the difference in fibrosis surrounding the bleb. In the present study, a ROCK inhibitor (ripasudil) was instilled before the needling procedure in one eye and other antiglaucoma medications were instilled in three eyes (prostaglandins [PG], one eye; beta-blockers, one eye; and alpha-2 agonists, one eye) in the control group. Seven eyes received the ROCK inhibitor and four eyes received other antiglaucoma medications (PG, two eyes; beta-blockers, one eye; and carbonic anhydrase inhibitors, one eye), which were instilled before the needling procedure in the ripasudil group (*p* = 0.035). In addition, 0.1% betamethasone was used only in the control group (6 eyes), whereas 0.1% fluorometholone was used in both groups (control group, 9 eyes; ripasudil group, 12 eyes, *p* = 0.013). Broadway et al. reported that the combination with antiglaucoma medications induced conjunctival inflammation before surgery, which is a risk factor for the failure of trabeculectomy [31, 32]. Miki et al. reported that the outcomes of trabeculectomy, especially the incidence of deepening of the upper eyelid sulcus (DUES) caused by PG, varied depending on the preoperative administration of PG [33]. Several studies have reported that the use of topical corticosteroids is associated with high anti-inflammatory potency and ocular side effects, which cause an increase in the IOP [34, 35]. Fluorometholone (0.1%) is a corticosteroid that has a reduced propensity for increasing IOP compared with 0.1% betamethasone. Therefore, the differences in the number and contents of antiglaucoma and anti-inflammatory medications may have contributed to the maintenance of or decrease in IOP. Further studies are needed to examine the influence of ROCK inhibitor on the bleb shape, meanwhile, the number and contents of antiglaucoma and anti-inflammatory medications should be matched to verify the efficacy of ROCK inhibitor after the needling procedure.

## Conclusions

Our study shows that the addition of the ROCK inhibitor ripasudil after the needling procedure with MMC did not improve surgical outcomes in terms of IOP. However, the addition of ripasudil may reduce the number of antiglaucoma medications in the midterm after the needling procedure with MMC. Further research with prospective clinical trials is required to reveal the role of ripasudil in antifibrosis after glaucoma surgery.

## Data Availability

The data analyzed in this study are available from the corresponding author upon reasonable request.

## Abbreviations

IOP: Intraocular pressure
5-FU: 5-Fluorouracil
MMC: Mitomycin C
ROCK: Rho-associated protein kinase
POAG: Primary open-angle glaucoma
PACG: Primary angle-closure glaucoma
G: Glaucoma
TLE: Trabeculectomy
MMC: Mitomycin C
IOL: Intraocular lens
PG: Prostaglandins
DUES: Deepening of the upper eyelid sulcus
